# Novel Influences of Sex and *APOE* Genotype on Spinal Plasticity and Recovery of Function after Spinal Cord Injury

**DOI:** 10.1523/ENEURO.0464-20.2021

**Published:** 2021-03-05

**Authors:** Lydia E. Strattan, Daimen R. S. Britsch, Chris M. Calulot, Rachel S. J. Maggard, Erin L. Abner, Lance A. Johnson, Warren J. Alilain

**Affiliations:** 1 Department of Neuroscience, University of Kentucky College of Medicine, Lexington, KY 40536; 2 Spinal Cord and Brain Injury Research Center, University of Kentucky College of Medicine, Lexington, KY 40536; 3 Sanders-Brown Center on Aging, University of Kentucky, Lexington, KY 40504; 4 Department of Biostatistics, University of Kentucky, Lexington, KY 40536; 5 Department of Epidemiology, University of Kentucky, Lexington, KY 40536; 6 Department of Physiology, University of Kentucky College of Medicine, Lexington, KY 40536

**Keywords:** apolipoprotein E, breathing, genetics, plasticity, spinal cord injury

## Abstract

Spinal cord injuries can abolish both motor and sensory function throughout the body. Spontaneous recovery after injury is limited and can vary substantially between individuals. Despite an abundance of therapeutic approaches that have shown promise in preclinical models, there is currently a lack of effective treatment strategies that have been translated to restore function after spinal cord injury (SCI) in the human population. We hypothesized that sex and genetic background of injured individuals could impact how they respond to treatment strategies, presenting a barrier to translating therapies that are not tailored to the individual. One gene of particular interest is *APOE*, which has been extensively studied in the brain because of its allele-specific influences on synaptic plasticity, metabolism, inflammation, and neurodegeneration. Despite its prominence as a therapeutic target in brain injury and disease, little is known about how it influences neural plasticity and repair processes in the spinal cord. Using humanized mice, we examined how the ε3 and ε4 alleles of *APOE* influence the efficacy of therapeutic intermittent hypoxia (IH) in inducing spinally-mediated plasticity after cervical SCI (cSCI). IH is sufficient to enhance plasticity and restore motor function after experimental SCI in genetically similar rodent populations, but its effect in human subjects is more variable (Golder and Mitchell, 2005; Hayes et al., 2014). Our results demonstrate that both sex and *APOE* genotype determine the extent of respiratory motor plasticity that is elicited by IH, highlighting the importance of considering these clinically relevant variables when translating therapeutic approaches for the SCI community.

## Significance Statement

There is currently a critical need for therapeutics that restore motor and sensory function effectively after cervical spinal cord injury (cSCI). Although many therapeutic approaches, including intermittent hypoxia (IH), are being investigated for their potential to enhance spinal plasticity and improve motor outcomes after SCI, it is unknown whether the efficacy of these treatment strategies is influenced by individuals’ genetic background. Here, we show that *APOE* genotype and sex both play a role in determining the propensity for motor plasticity in humanized mice after cervical SCI (cSCI). These results indicate that sex and genetic background dictate how individuals respond to therapeutic approaches, thereby emphasizing the importance of developing personalized medicine for the diverse SCI population.

## Introduction

Over 17,000 Americans experience a spinal cord injury (SCI) every year (National Spinal Cord Injury Statistical Center, 2018). Depending on the level of injury, damage to neural pathways in the spinal cord can lead to a multitude of sensory deficits and loss of crucial motor functions. Over the past few decades, many promising therapeutic approaches have been developed to enhance neuroprotection or induce anatomic and functional plasticity of spinal pathways to restore function (Fuller et al., 2003; Huie et al., 2017; Satkunendrarajah et al., 2018; Zholudeva et al., 2018; Jack et al., 2020). Moreover, pivotal studies using nerve grafts, PTEN deletion, NOGO inhibition, or degradation of the perineuronal net or chondroitin sulfate proteoglycans (CSPGs) have demonstrated that the CNS is capable of overcoming neural intrinsic and extrinsic barriers to regeneration after injury, leading to meaningful preclinical recovery (David and Aguayo, 1981; Chen et al., 2000; Park et al., 2008; Alilain et al., 2011; Urban et al., 2019). However, these therapeutic strategies have met with varied clinical success and there remains a lack of effective treatment strategies for the human SCI population (for review, see Ahuja et al., 2017).

A striking difference between individuals living with SCI and the animals used to model them is the level of genetic diversity represented in these populations. In contrast to the incredible diversity of the human population, preclinical studies typically use homogenous groups of animals with the same sex and similar genetic backgrounds. While this does facilitate easier determination of treatment effects, it also makes it less likely that discoveries in these models will translate to human patients. Although an increasing number of preclinical investigations are addressing how sex influences the efficacy of therapeutic strategies, the impact of genetic variability remains largely unexplored. A recent review by Fouad et al. (2020), specifically outlined the importance of evaluating the influence of factors such as sex and genotype to address the neuroanatomical-functional paradox and lack of therapeutic translation in SCI. Indeed, we hypothesize that genetic factors could play a considerable role in determining how individuals respond to treatment strategies.

Apolipoprotein E (ApoE) is a highly expressed lipid carrier in the CNS (Boyles et al., 1985). It is encoded by the *APOE* gene, which exists in three common alleles designated ε2, ε3, and ε4. The ε4 allele, which is carried by nearly one in five individuals, has been associated with a number of detrimental outcomes, including a weakening of synaptic plasticity in the brain (Zhao et al., 2018). However, despite a robust body of literature in neurodegenerative diseases and traumatic brain injury (Zhou et al., 2008; Mahley, 2016; Main et al., 2018); the impact of ε4 on plasticity in the spinal cord remains underexplored. We hypothesized that spinally-mediated plasticity is constrained in apoE4 animals, thereby demonstrating the importance of considering the diversity of the human population when developing therapeutic approaches for people with SCI.

To test this hypothesis, we use a model of cervical injury to examine how *APOE* genotype alters the response to intermittent hypoxia (IH). Most SCIs occur at these high levels and can disrupt the neural circuitry that mediates breathing, leading to respiratory insufficiency and potentiating the need for mechanical ventilation (Bergofsky, 1964; Alp and Voss, 2006; National Spinal Cord Injury Statistical Center, 2018). Mechanical ventilation increases the risk of respiratory infection, a leading cause of rehospitalization and death following cervical SCI (cSCI; DeVivo and Ivie, 1995).

In recent years, there has been a growing appreciation for the potential of IH as a treatment strategy for a host of conditions including SCI (Navarrete-Opazo and Mitchell, 2014). In clinical trials, therapeutic IH has been used to increase limb function and to facilitate ventilation in persons with SCI by enhancing plasticity in the spinal cord (Tester et al., 2014; Lynch et al., 2017; Trumbower et al., 2017). Neural pathways in the cervical region which mediate breathing are critical therapeutic targets of IH, including spared pathways which might remain after injury. However, the influence of human genetic variability on IH-induced recovery is unknown. Therefore, we used this model of spinally-mediated plasticity to examine how expression of different human *APOE* alleles alter the efficacy of therapeutic strategies, such as IH, that are being developed to enhance plasticity following SCI. Our results provide evidence that both sex and *APOE* genotype determine the propensity for plasticity in humanized mice that are exposed to therapeutic IH.

## Materials and Methods

### C2 hemisections

All experiments were approved by the Institutional Animal Care and Use Committee at the University of Kentucky. Mice expressing human *APOE* isoforms under control of the mouse *APOE* promotor (targeted replacement mice) were backcrossed for at least 10 generations to the C57BL/6 background (Sullivan et al., 1997, 1998; Knouff et al., 1999). Mice were group-housed on a 12/12 h light/dark cycle and fed normal chow diet *ad libitum.* All mice were 92–105 d old at the time of injury. Female (20–24 g) and male (22–30 g) mice were anesthetized with isoflurane. Animals were then prepped for surgery by shaving the surgical area followed by disinfecting with alternating betadine and 70% ethanol swabs. Puralube ophthalmic ointment was applied to the eyes to prevent drying during surgery. A midline incision was made through the skin just caudal to the ears to between the scapulae. Marcaine/bupivacaine was instilled along the incision site. The acromiotrapezius, semispinalis capitus, and rectus capitus posterior muscles were cut along their midline, bluntly dissected, and retracted. Paravertebral muscles were cut away from the C2 vertebra using ToughCut Spring Scissors (Fine Science Tools). The lamina of the C2 vertebra was then removed using Spring Scissors. Under a dissecting microscope (Meiji EMZ), a left lateral C2 hemisection (C2Hx) was performed by inserting a 27-gauge needle into the midline of the spinal cord at the C2 level and dragging the needle to the left lateral edge of the cord. This was then repeated once to ensure a complete injury. Musculature was sutured (6–0 absorbable suture) and skin was closed with Vetbond Tissue Adhesive (3 M). Animals received subcutaneous buprenorphine (0.75 mg/kg) and carprofen (10 mg/kg) immediately after surgery. Male mice were housed individually following surgery to prevent fighting among cage mates.

### IH and diaphragmatic electromyography (EMG)

**Figure 1.**
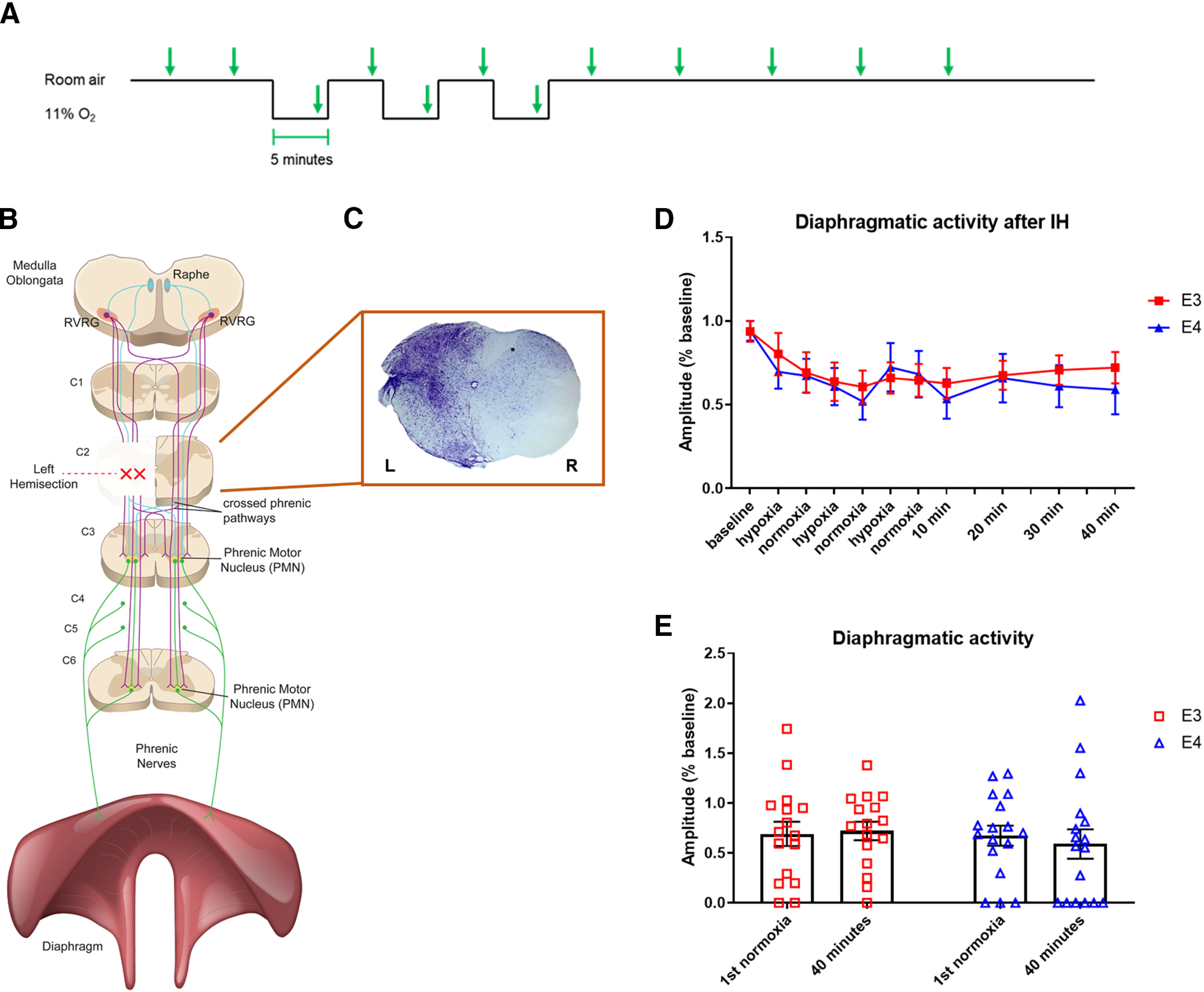
Magnitude of respiratory motor plasticity is not determined by *APOE* genotype alone. ***A***, Timeline of IH protocol. Green arrows represent time points at which peak amplitude was analyzed. ***B***, Schematic of the neural circuitry that mediates breathing. Location of the left C2 hemisection is indicated by red X. ***C***, Representative image of cresyl violet staining of the spinal cord at the C2 level after injury. ***D***, Quantification of diaphragmatic amplitude over time during and after IH. There was no difference between genotypes in the change in amplitude over time (*a.* RMANOVA *p* = 0.741, MD = 0.043, CI = −0.22−0.31) ***E***, Quantification of diaphragmatic amplitude during the first normoxic bout and 40 min after IH (*b.* paired *t* test E3 normoxia v. 40 min *p* = 0.741, MD = 0.029, CI = −0.16−0.22; *c.* E4 normoxia v. 40 min *p* = 0.405, MD = 0.084, CI = −0.29−0.12). Bars show mean and SEM values.

Three weeks after hemisection, animals were anesthetized with isoflurane using the SomnoSuite Anesthesia System (Kent Scientific). A laparotomy was performed by cutting through the rectus abdominis, external oblique, and internal oblique muscles. Bipolar electrodes, connected to an amplifier and data acquisition system (CWE BMA-400 Four-channel Bioamplifier, CED 1401 with Spike2 Data Analysis Computer Interface), were inserted into the dorsal region of the left hemidiaphragm, where they were secured using Vetbond. Bilateral recordings were not performed because of the increased attrition rate we observed after performing bilateral electrode insertion. The laparotomy was also closed using Vetbond; 10 min of baseline breathing activity was recorded. The air input to the Somnosuite was then changed from room air (normoxia) to a tank of 11% oxygen, 89% nitrogen gas (hypoxia) for 5 min, at which point it was switched back to room air for 5 min. This was repeated for three bouts of hypoxia separated by 5 min of normoxia. Diaphragmatic activity was recorded for 1 h after the final hypoxic bout (Fig. 1*A*). Although core temperature was not monitored during recordings, animals were kept on heating pads throughout all recording procedures to maintain body temperature.

### Sectioning and staining

To harvest tissue for immunohistochemistry, mice were perfused with 4% paraformaldehyde (PFA) following the diaphragmatic EMG recording. The spinal column was isolated and placed in 4% PFA at 4°C. After 2 d, tissue was removed from PFA and placed in a 30% sucrose solution at 4°C for cryoprotection until sectioning.

Tissue was mounted and frozen in Tissue Plus O.C.T. Compount (Fisher Healthcare) and cut at a thickness of 20 μm on a cryostat (Leica). Serial sections from the injury site (C1–C2) were placed on one set of slides while serial sections from the level of the PMN (C3–C6) were placed on another set. Injured tissue slides were dehydrated in ethanol and stained with 0.1% cresyl violet solution (Sigma catalog #C5042). Slides were then mounted using permount (Electron Microscopy Sciences catalog #17986-01). For 5-HT staining, frozen sections were thawed to room temperature, rinsed with 1× PBS, and blocked in a solution of 5% normal goat serum (NGS), 0.1% bovine serum albumin, and 0.1% Triton X-100 dissolved in PBS. Slides were incubated in 5-HT primary antibody diluted 1:10,000 (rabbit, ImmunoStar catalog #20080) then goat anti-rabbit Alexa Fluor 488 secondary antibody (1:500, Life Technologies catalog #A11034). Stained slides were mounted with ProLong Gold mountant with DAPI (Invitrogen catalog #P36931). For *Wisteria floribunda* lectin (WFA) staining, frozen sections were thawed to room temperature, washed with 1× PBS, then blocked in 3% NGS diluted in PBS. Slides were then incubated in WFA primary antibody conjugated to Fluorescein at a dilution of 1:400 (Vector Labs catalog #FL-1351). Stained slides were mounted in ProLong Gold with DAPI.

### Trace analysis

After recording, raw diaphragmatic EMG was rectified and integrated using Spike2 software. Analysis was performed at twelve time points: twice during baseline recording, once during each hypoxic and normoxic bout, and at 10, 20, 30, and 40 min after the final hypoxic period (Fig. 1*A*). For each time point, peak amplitude was averaged over a 30-s period. Amplitude of diaphragmatic bursts at each time point were normalized to that animal’s prehypoxia baseline amplitude. Frequency of diaphragmatic bursts, indicative of breaths, was also quantified over a 30-s period at each time point.

### Imaging and image quantification

Staining for cresyl violet and WFA was imaged on a Keyence BZ-X810 fluorescence microscope for quantification. Cresyl violet-stained sections were imaged using brightfield illumination at 2×. Sections stained for WFA were imaged at 10× using the monochromatic camera with high resolution (0.75488 μm/pixel) for quantification. Additional images for publication were acquired on a Nikon Eclipse T*i* series inverted confocal at 40×, focused on the ventral horn in the region of the putative PMN. Sections stained for 5-HT were imaged on the Nikon at 20×. Images of 5-HT staining for publication were taken at 40× in the region of the PMN. All imaging and quantification were performed on the ventral horn of the left side of the cord, ipsilateral to the injury.

WFA labeling was quantified using the HALO image analysis platform (Indica Labs). We developed and optimized an algorithm on the Area Quantification v1.0 to capture positive staining for WFA while omitting any nonspecific fluorescence. A region of interest (ROI) was drawn around the left ventral horn of sections at the level of C4. The quantification algorithm was applied to the ROI of each section. The area of staining was then normalized to the total area of the ROI. Three tissue sections at level C4 were analyzed for each animal. 5-HT labeling was also quantified with HALO. A ROI was drawn around the left ventral horn. Serotonergic fibers within the ROI were traced using the embedded annotation tool. The total length of fibers was then normalized to the area of the ROA.

### Experimental design and statistical analyses

Sample sizes for mice receiving diaphragmatic EMG recordings were calculated based on preliminary data from 10 hemisected mice representing all three genotypes using Cohen’s *d* to measure effect size. Group sizes for each sex and genotype are found in Table 1. Tissue from a subset of animals was perfused with PFA and spinal cord tissue was harvested from these animals for IHC and quantification of WFA and serotonergic sprouting (apoE3 *n* = 4, apoE4 *n* = 5).

**Table 1:** Group sizes for diaphragmatic EMGs

	**Male**	**Female**
E3	*n* = 7	*n* = 8
E4	*n* = 6	*n* = 11

For statistical analysis of EMG traces, repeated measures (RM)ANOVA was used to account for within-subject correlation given repeated measurements over time. Stratified RMANOVA analyses were performed on male and female traces. Results were considered statistically significant if *t* ≥ 1.96. Student’s *t* test was used to analyze 5-HT fiber staining on spinal cord tissue. Welch’s *t* test for unequal variances was used to analyze staining of WFA. Results were considered statistically significant if *p* < 0.05. Investigators were blinded until all diaphragmatic EMG and histology analyses were complete. The mean difference (MD) and 95% confidence interval (CI) were calculated to provide an estimate of the range of possible differences between groups (Table 2).

**Table 2:** Statistical tests

Data structure	Type of test	95% CI
*a*. Repeated measurements, normal distribution	RMANOVA	−0.2202 to 0.3063
*b*. Normal distribution with within-subject correlation	Paired *t* test	−0.1570 to 0.2160
*c*. Normal distribution with within-subject correlation	Paired *t* test	−0.2907 to 0.1235
*d*. Repeated measurements, normal distribution	RMANOVA	−0.2958 to 0.35575
*e*. Repeated measurements, normal distribution	RMANOVA	−1.17798 to 0.07798
*f*. Repeated measurements, normal distribution	RMANOVA	−1.53798 to −0.2820
*g*. Repeated measurements, normal distribution	RMANOVA	−0.9958 to −0.3442
*h*. Repeated measurements, normal distribution	RMANOVA	−0.75238 to 0.11238
*i*. Repeated measurements, normal distribution	RMANOVA	0.426832 to 1.25317
*j*. Repeated measurements, normal distribution	RMANOVA	1.66683 to 2.49317
*k*. Repeated measurements, normal distribution	RMANOVA	0.64132 to 1.37868
*l*. Normal distribution, Unequal variance	Welch’s *t* test	−0.0003607 to 0.006133
*m*. Normal distribution	Student’s *t* test	0.0003556 to 0.002912
*n*. Normal distribution	Student’s *t* test	−0.001091 to 0.003178
*o*. Normal distribution	Student’s *t* test	−0.2916 to 1.237

## Results

### Respiratory motor plasticity in C2 hemisected humanized *APOE* mice

At three months of age, male and female mice received a left C2 hemisection by making an incision from the midline to the left lateral edge of the spinal cord just caudal to the C2 dorsal roots. This injury effectively disrupts the neural circuitry that descends from the ipsilateral medullary respiratory nuclei to phrenic motor neurons on the left side (Fig. 1*B*). At the time of injury, hemisection was visually confirmed by observing the thorax of each mouse to ensure that only the right side of the thorax continued rhythmically expanding with each breath. Injury completeness was histologically confirmed on killing of a subset of mice (*n* = 16) using cresyl violet (Fig. 1*C*). All mice were homozygous for human ε3 or ε4 alleles expressed under control of the murine *APOE* promoter as described previously (Sullivan et al., 1997; Knouff et al., 1999). At three weeks postinjury, mice were exposed to IH. This consisted of 3 hypoxic (11% O_2_) bouts of 5-min duration separated by 5 min of normoxia as illustrated in Figure 1. We evaluated the breathing response to IH by concurrently recording diaphragmatic EMG, which continued for 1 h following the final hypoxic bout. Amplitude of diaphragmatic bursts was quantified while blinded and then grouped according to *APOE* genotype. No difference was found in the response to IH between mice expressing ε3 or ε4 (RMANOVA *p* = 0.741; Fig. 1*D*). All animals appear to experience an initial decrease in diaphragmatic activity during the first hypoxic bout. Breathing in both apoE3 and apoE4 mice remained constant once the IH protocol ended (Fig. 1*D*).

Previous studies in rodents (Bach and Mitchell, 1996; Baker and Mitchell, 2000; for review, see Fuller et al., 2000; Terada et al., 2008) have shown that IH treatment gives rise to an augmentation of breathing activity that characterizes long term facilitation (LTF). We therefore compared amplitude at the beginning of IH and 40 min after the final bout of hypoxia to determine whether breathing activity increased in response to IH. Neither genotype exhibited significant augmentation of diaphragmatic activity at 40-min posthypoxia, indicative of a lack of LTF in the humanized mice (paired *t* test E3 *p* = 0.741, E4 *p* = 0.405; Fig. 1*E*).

### Sex differences in apoE modulation of LTF

**Figure 2.**
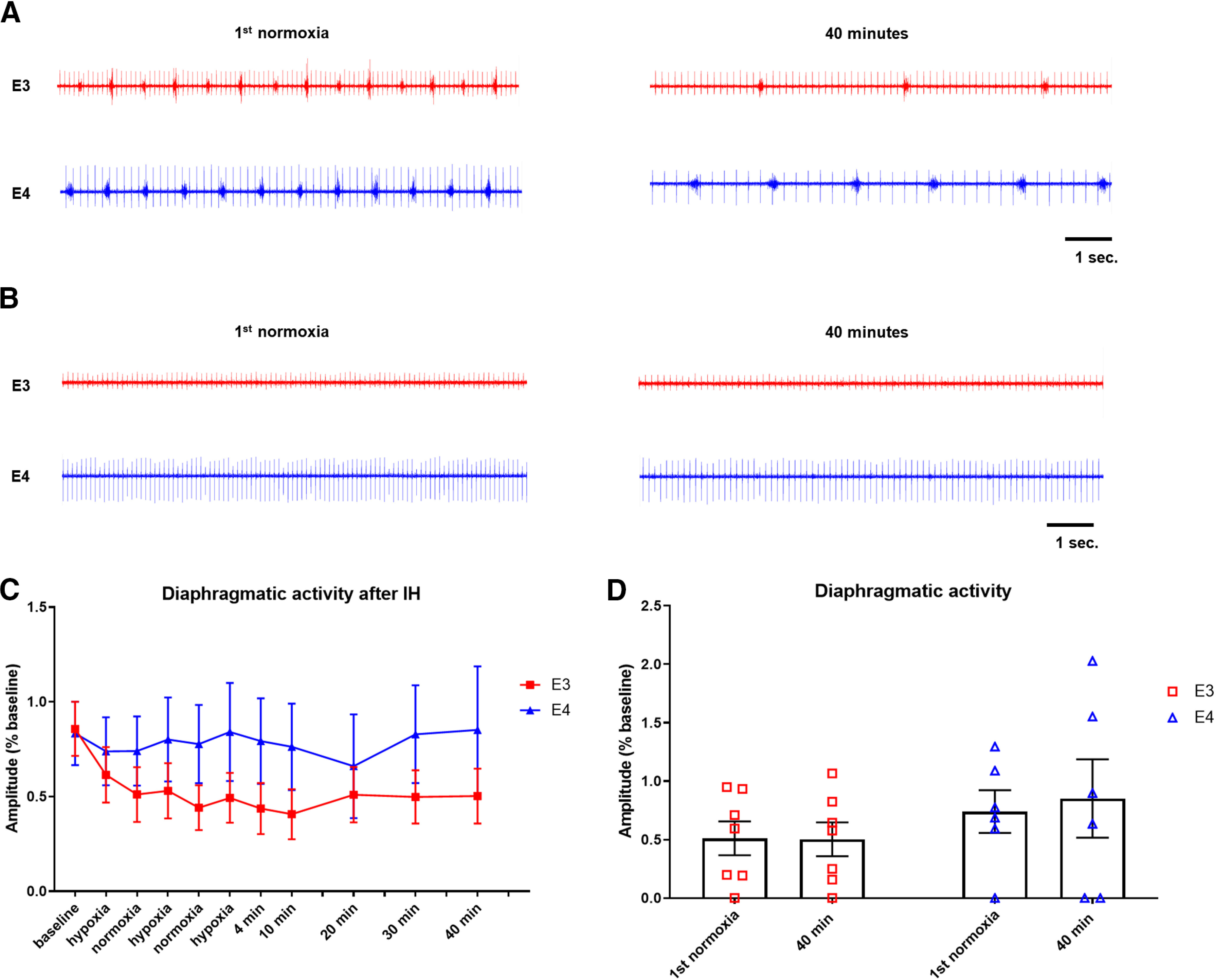
ApoE3 males demonstrate a trend of decreasing diaphragmatic activity in response to IH. ***A***, Representative traces of diaphragmatic activity during the first normoxic bout and 40 min after IH. ***B***, Representative traces from male mice that had no spontaneous recovery. ***C***, Quantification of diaphragmatic activity over time during and after IH. Amplitude of diaphragmatic bursts is not significantly different between E3 and E4 animals. ***D***, Quantification of diaphragmatic activity during the first normoxic bout and 40 min after IH (*d.* RMANOVA E3/E3 *t* = 0.03, MD = 0.0086, CI = −0.295−0.36; *e.* E3/E4 norm *t* = −0.55, MD = 0.23, CI = −1.18−0.078; *f.* E3/E4 40 min *t* = −0.91, MD = 0.35, CI = −1.54 to −0.28; *g.* E4/E4 *t* = −0.67, MD = 0.11, CI = −0.41−0.63). Bars show mean and SEM values.

To investigate sex-dependent influences of *APOE* genotype on LTF, we separated data from males and females for independent analysis. Animals were weighed every day for the first 4 d after injury and then once a week until diaphragmatic EMG recordings were performed. When comparing weights over time after injury, there was no significant difference between genotypes in male (*p* = 0.16) or female (*p* = 0.65) mice (data not shown). Figure 2*A* shows representative traces from male apoE3 and E4 mice. As evidenced in these traces, both genotypes exhibited a decrease in frequency over time after IH. However, there was no significant genotype effect on the magnitude of this decrease (RMANOVA, *p* = 0.846; Fig. 2*A*; Extended Data Fig. 2-1*A*). Previous studies in rats (Warren et al., 2018) have reported no spontaneous recovery in the paralyzed hemidiaphragm even chronically after C2 hemisection. In contrast, the overwhelming majority of the 32 mice used in the current study showed spontaneous functional recovery of the paralyzed mouse hemidiaphragm. Considering all males and females from which we recorded diaphragmatic EMGs, only two mice displayed no spontaneous recovery: one male of each genotype (Fig. 2*B*). Quantification of the diaphragmatic EMG data demonstrates that males expressing the ε3 allele display a decline in the amplitude of diaphragmatic bursting beginning in the first hypoxic bout and persisting throughout the recording period (Fig. 2*C*). Although this deterioration of activity did not reach statistical significance (RMANOVA, *t* = 0.03), it is worthwhile to highlight how the apoE3 males diverged from apoE4, which demonstrate slightly heightened activity at 40 min post-IH (RMANOVA, *t* = −0.65; Fig. 2*C*,*D*).

10.1523/ENEURO.0464-20.2021.f2-1 Extended Data Figure 2-1 The respiratory response to hypoxia is determined by *APOE* genotype in male mice. ***A***, Quantification of diaphragmatic burst frequency in male mice. There is no significant difference between the decreases in apoE3 and apoE4 mice (RMANOVA *p* = 0.846). ***B***, ***C***, Quantification of diaphragmatic burst amplitude (***B***) and frequency (***C***) in response to a 10-min hypoxic exposure. Hypoxia appears to attenuate breathing in apoE3 males. No statistics were performed due to low *n*; E3 and E4 *n* = 2. Download Figure 2-1, TIF file.

**Figure 3.**
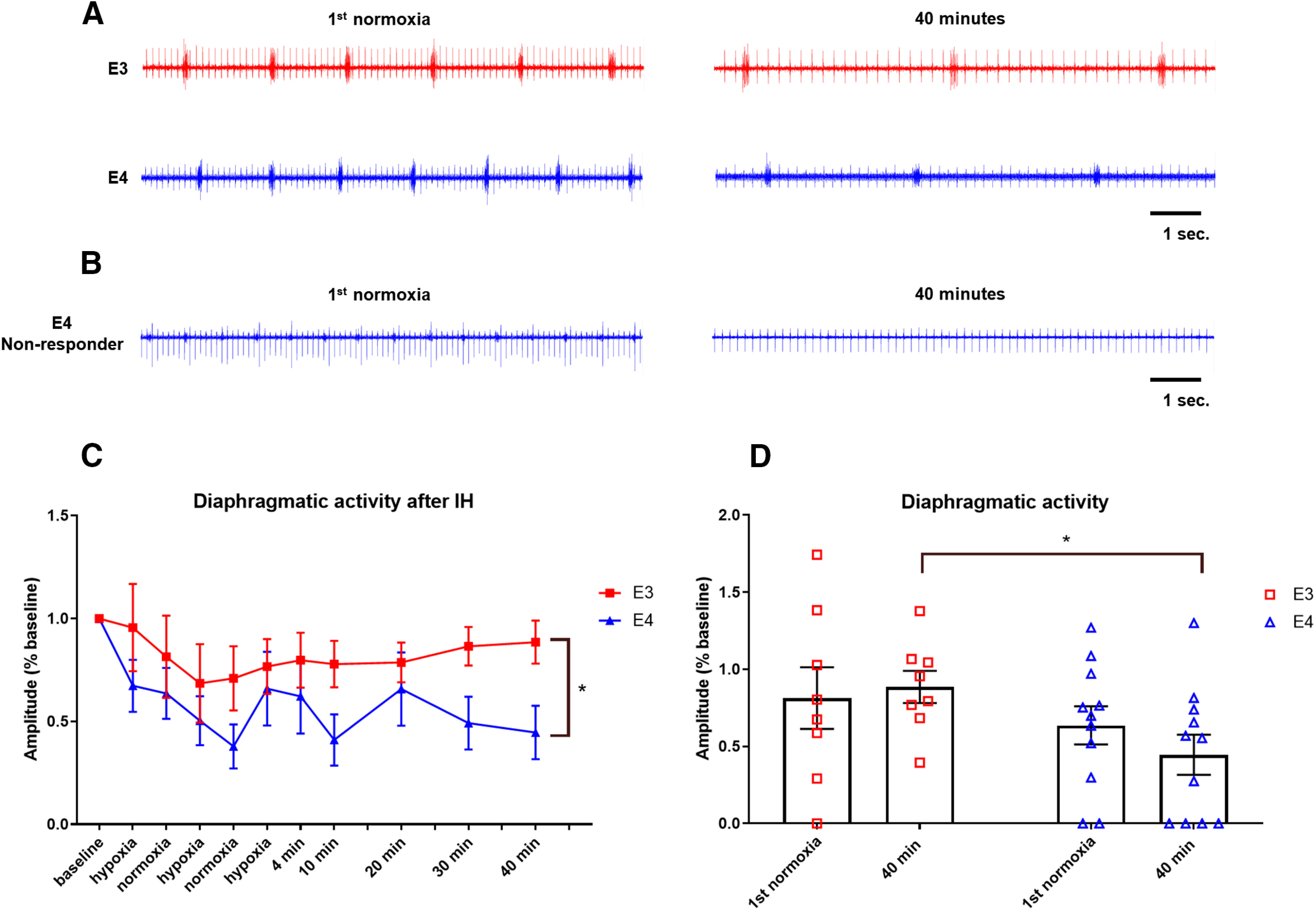
ApoE4 females display significantly less diaphragmatic activity than E3 females after IH. ***A***, representative traces of diaphragmatic activity during the first normoxic bout and 40 min after IH. ***B***, Representative traces from an apoE4 female non-responder. ***C***, Quantification of diaphragmatic activity over time during and after IH. Amplitude of diaphragmatic bursts are significantly greater in E3 females than in E4. ***D***, Quantification of diaphragmatic activity during the first normoxic bout and 40 min after IH (*h.* RMANOVA E3/E3 *t* = −0.32, MD = 0.071, CI = −0.75−0.11; *i.* E3/E4 normoxia *t* = 0.84, MD = 0.18, CI = 0.43–1.25; *j.* E3/E4 40 min *t* = 2.08, MD = 0.44, CI = 1.67–2.49; *k.* E4/E4 *t* = 1.01, MD = 0.19, CI = 0.64–1.38). Asterisk represents statistical significance (*t* >1.96) Bars show mean and SEM values.

Analysis of diaphragmatic EMGs in female mice of both genotypes showed a similar negative trend in the breathing frequency induced by IH (RMANOVA, *p* = 0.673; Fig. 3*A*; Extended Data Fig. 3-1). A subset of mice displayed a complete loss of diaphragmatic activity following hypoxic exposure. We refer to these animals as “non-responders.” Three non-responders emerged in the apoE4 group, while none were observed in the mice that expressed ε3 (representative trace shown in Fig. 3*B*). However, unlike the male mice, all females demonstrated spontaneous recovery before IH (data not shown). Quantification of diaphragmatic burst amplitude in females that maintained diaphragmatic activity after IH showed that apoE3 mice responded to IH with an initial decrease in burst amplitude. This decline was temporary and activity returned to near baseline levels by 40 min (Fig. 3*C*). However, apoE4 females exhibited an immediate reduction in burst amplitude that is still evident the end of the recording period. At 40 min post-IH, breathing of apoE4 females is significantly depressed compared with that of apoE3’s at the same time point (mixed model RMANOVA *t* = 2.08; Fig. 3*C*,*D*). Consistent with the combined data, none of the female mice expressing human *APOE* developed the gradual and prolonged breathing augmentation that is characteristic of LTF.

10.1523/ENEURO.0464-20.2021.f3-1 Extended Data Figure 3-1 Hypoxia induces a decline in breathing frequency in female APOE targeted replacement mice. ***A***, Quantification of diaphragmatic burst frequency in female mice. There is no significant difference between the decreases in apoE3 and apoE4 mice (RMANOVA *p* = 0.673). ***B***, ***C***, Quantification of diaphragmatic burst amplitude (***B***) and frequency (***C***) in response to a 10-min hypoxic exposure. Breathing frequency displayed a negative trend in apoE4 females. No statistics were performed due to low *n*; E3 *n* = 3, E4 *n* = 2. Download Figure 3-1, TIF file.

When animals are challenged with a brief bout of hypoxia, feedback from peripheral chemoreceptors induces an augmentation of respiratory output. This change in ventilation is known as the hypoxic ventilatory response (HVR; described by Pamenter and Powell, 2016). During the hypoxic bouts of IH treatment, all apoE mice exhibited a decline in diaphragmatic activity instead of the expected amplification. To further investigate the HVR in our humanized mice, we exposed an additional, smaller cohort of mice to a 10-min bout of hypoxia and assessed the changes in amplitude and frequency of diaphragmatic bursting. No females of either genotype displayed an increase or decrease in amplitude, but breath frequency began to decline by the end of the hypoxic period in those expressing ε4 (Extended Data Fig. 3-1*B*,*C*). In male mice expressing ε3, there was a sharp decline in both amplitude and frequency of diaphragmatic firing in response to hypoxia such that breathing activity was abolished at 10 min. Conversely, amplitude and frequency in apoE4 males remained constant during hypoxia (Extended Data Fig. 2-1*B*,*C*).

### Perineuronal net upregulation and serotonergic sprouting in the phrenic motor nucleus

**Figure 4.**
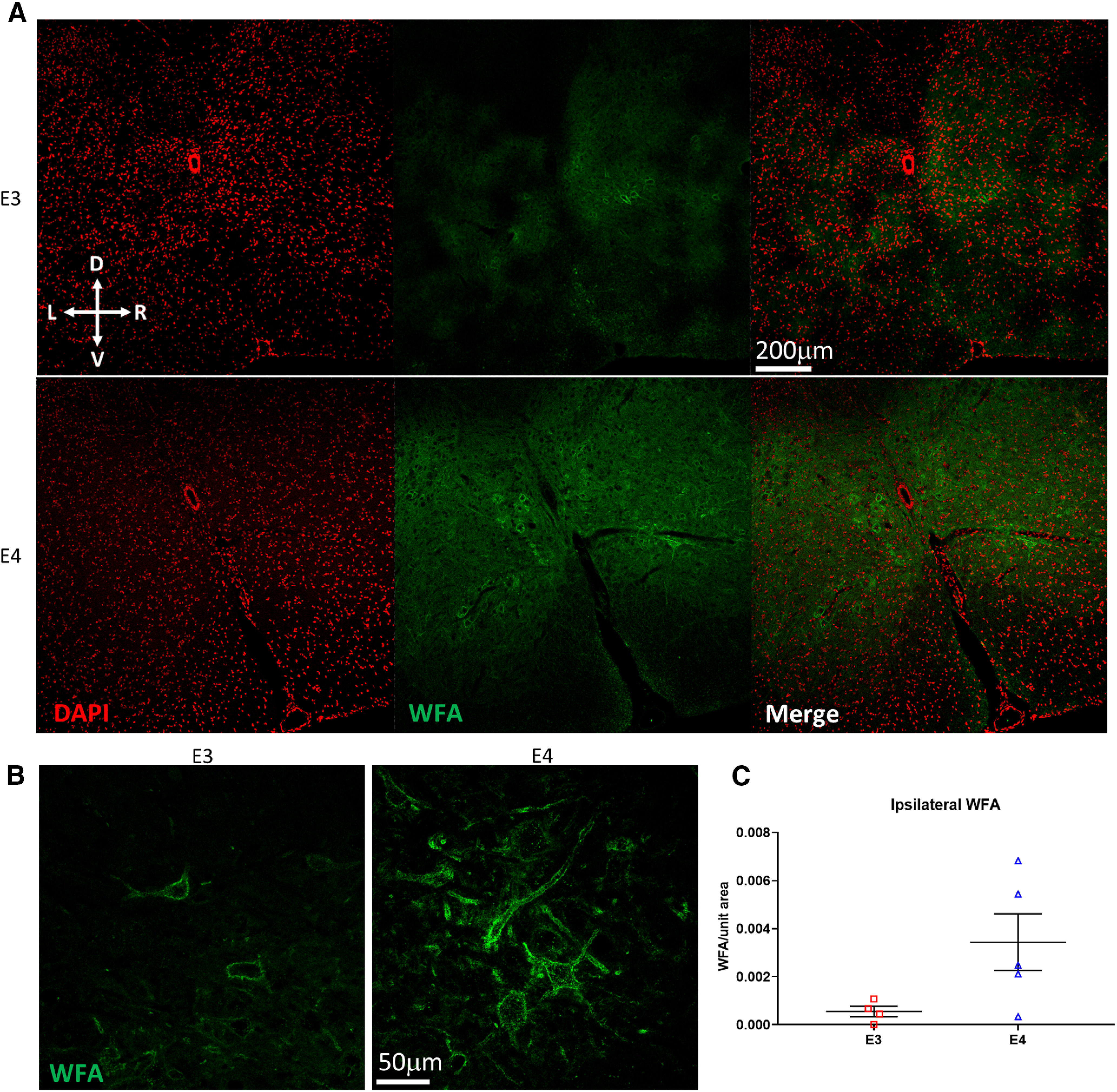
E4 females have higher levels of PNN. ***A***, Representative images of WFA staining at the C4 spinal cord level (DAPI is in red, WFA is in green). ***B***, Higher-magnification images show the PNN surrounding putative phrenic motor neurons. ***C***, Quantification of WFA indicates that apoE4 mice express more WFA than E3 mice, although this trend is not statistically significant (*l.* Welch’s *t* test *p* = 0.0697, MD = 0.0029, CI = −0.00036−0.0061). Bars represent mean ± SEM.

Secretion of CSPGs, a component of the perineuronal net (PNN), is upregulated after SCI in wild-type animals, creating a barrier to plasticity, regeneration, and sprouting (Tom et al., 2009; Alilain et al., 2011). Thus far, it is unknown whether the magnitude of this upregulation is modulated by human *APOE* genotype. Therefore, we used WFA staining to compare the amount of PNN present in injured spinal cords at the C4 level to determine whether the IH-induced reduction in diaphragmatic activity observed in E4 females was correlated with increased amounts of inhibitory PNN. Indeed, we found that apoE4 females tended to have a higher density of WFA around the phrenic motor nucleus after injury, although this trend did not reach significance (Welch’s *t* test, *p* = 0.0697; Fig. 4*A–C*).

**Figure 5.**
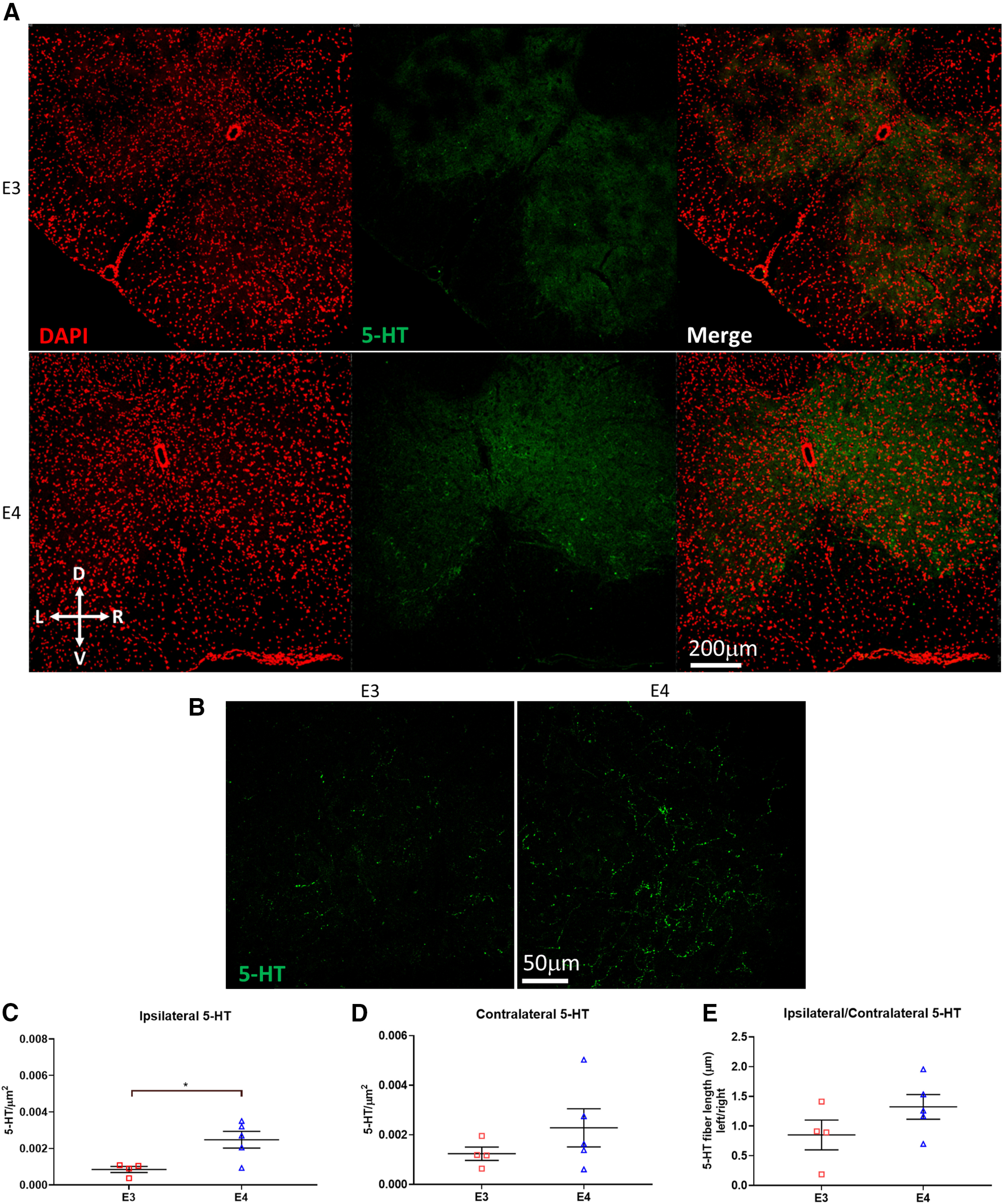
E4 females have higher density of spinal 5-HT fibers. ***A***, Representative images of stained 5-HT fibers in the C4 spinal cord level. ***B***, Higher magnification shows individual 5-HT fibers in the area of the putative PMN. ***C***, Significantly more serotonergic fibers are found ipsilateral to injury in apoE4 females (*m.* Student’s *t* test *p* = 0.0193, MD = 0.0016, CI = 0.00036−0.0029). ***D***, 5-HT fibers contralateral to injury at the C4 level (*n*. Student’s *t* test *p* = 0.286, MD = 0.00,104, CI = −0.0011−0.0032). ***E***, Ipsilateral 5-HT staining normalized to contralateral (*o.* Student’s *t* test *p* = 0.187, MD = 0.47, CI = −0.29–1.24). Asterisk represents statistical significance (*p* < 0.05). Bars represent mean ± SEM.

The PNN can limit 5-HT sprouting after injury (Alilain et al., 2011). To determine whether differences in respiratory motor plasticity observed in females were because of the amount of serotonin at the level of the phrenic motor nucleus after C2Hx, serotonergic fibers were labeled and quantified in the ventral horn ipsilateral to injury. Serotonergic sprouting after injury has previously been correlated with the restoration of breathing function and enhancement of LTF (Golder and Mitchell, 2005). We postulated that dampened respiratory plasticity in ε4 females may be because of a lack of serotonergic sprouting after injury. Surprisingly, quantification of 5-HT+ fibers ipsilateral to injury revealed enhanced serotonergic sprouting in apoE4 females compared with apoE3 (Student’s *t* test, *p* = 0.0193; Fig. 5*A–C*). This contradicted our expectation that a blunted respiratory response to IH would correspond with attenuated fiber sprouting after injury. Quantification of 5-HT fibers contralateral to injury showed no significant difference between E3 and E4 females, although E4 tended to have more 5-HT staining (Student’s *t* test, *p* = 0.286; Fig. 5*D*). After injury, E4 females had more 5-HT fibers ipsilateral than contralateral to injury, although this did not reach statistical significance (Student’s *t* test, *p* = 0.187; Fig. 5*E*).

## Discussion

This study represents the first investigation into human genetic influences on the efficacy of experimental therapeutic strategies for SCI. Our results demonstrate that individuals’ propensity for initiating beneficial neuroplastic responses to therapeutic IH is modified by sex and *APOE* genotype. By using a well-described model of SCI and spinally-mediated motor plasticity, we provide evidence to support the hypothesis that human genetic factors that are not represented by preclinical animal models limit the potential for recovery after SCI. Our physiology and histology data indicate that sex and genotype influence the CNS response to injury and therapeutic intervention, which poses a significant challenge to translating one-size-fits-all treatment strategies.

### *APOE* genotype and respiratory motor plasticity

Recovery of breathing function is a top priority for people living with cSCI (Anderson, 2004). IH has promising potential to enhance spinal plasticity for the restoration of a variety of motor behaviors, including breathing (Fuller et al., 2003; Lovett-Barr et al., 2012; Trumbower et al., 2012). Studies by Wadhwa et al. (2008) and Tester et al. (2014) in human participants have demonstrated that ventilatory LTF is expressed by both male and female subjects, even when living with a chronic SCI. However, to our knowledge, the interaction of sex and genetic factors remains unexplored in the LTF literature. Preclinical studies that have addressed the impact of sex on respiratory motor plasticity revealed that sex hormone levels have significant ramifications for the potential to induce plasticity, likely because of the interaction of sex hormones and the serotonergic system (Zabka et al., 2001a,b, 2003). Additionally, Baker-Herman et al. (2010) found that rat strains of different genetic backgrounds vary in their responses to IH, which was associated with differences in the expression of 5-HT_2A_ receptors on PMNs.

To further address how genetic variability impacts spinal plasticity, we examined the efficacy of IH for inducing LTF in targeted replacement mice expressing the human apoE ε3, and ε4 alleles. Since apoE first gained notoriety as a genetic marker for Alzheimer’s disease (AD), an extensive body of literature has investigated the impact of the apoE isoforms in the brain. The ε4 allele increases the risk of developing AD and lowers the age of onset in a dose-dependent manner (Corder et al., 1993; Saunders et al., 1993). E4 carriers display mitochondrial dysfunction, aggravated neuroinflammatory responses to CNS damage, loss of blood brain barrier integrity, and impaired synaptic plasticity (Safieh et al., 2019). These factors are also key determinants for the extent of tissue damage, plasticity, regeneration, and the potential for recovery after SCI (Noble and Wrathall, 1989; Sullivan et al., 2007; Alilain and Goshgarian, 2008; Kigerl et al., 2009).

ApoE further became a gene of interest in our investigation after studies in human SCI patients found that people who carried the ε4 allele experienced significantly less motor recovery than non-carriers, despite spending more time in rehabilitation (Jha et al., 2008; Sun et al., 2011). ApoE4 is known to curb recycling of NMDA and AMPA receptors to the postsynaptic membrane and reduces levels of BDNF in the CNS (Chen et al., 2010; Chhibber and Zhao, 2017; Sen et al., 2017). Since LTF requires BDNF signaling and activation of NMDA receptors, individuals expressing the ε4 allele may have a constrained response to IH (Baker-Herman et al., 2004; McGuire et al., 2005). However, our data demonstrates that mice expressing human apoE isoforms did not differ in their diaphragmatic response to IH, indicating that there may be no effect on LTF when *APOE* genotype is the sole variable being considered.

The lack of divergence between genotypes and the absence of augmented diaphragmatic activity in response to IH could also be because of metabolic changes. Many protocols for the induction of LTF, which are primarily conducted in rats, include the measurement of pCO_2_ throughout recording (Hayashi et al., 1993; Bach and Mitchell, 1996). This measurement provides a gauge of how metabolism is changing as a result of hypoxic hypometabolism: instead of increasing respiratory activity, small mammals respond to hypoxic conditions by downregulating their metabolic rate to reduce oxygen consumption (Hill, 1959). Because we did not measure pCO_2_ during EMG recordings because of the low blood volume of mice, we were unable to control for changes in metabolic rate, which could have prevented IH-induced breathing augmentation. However, we do not think that hypometabolism was responsible for masking genotype effects since differences emerged when animals were grouped according to sex.

Interestingly, mice displayed a decrease in ipsilateral hemidiaphragmatic activity during hypoxic bouts, instead of the heightened activity that is typical of the HVR observed in rats (Pamenter and Powell, 2016). Very little data are available on the respiratory response to IH and manifestation of LTF in C2 hemisected mice, although Minor et al. (2006) demonstrated the presence and viability of the murine crossed phrenic pathway (CPP), the anatomic substrate that mediates LTF (Golder and Mitchell, 2005). The few studies performed in mice are variable in IH protocols and methods of assessing LTF (Terada et al., 2008; Hickner et al., 2014; ElMallah et al., 2016). Our HVR data indicates that mice respond to bouts of hypoxia differently than rats, but additional experiments are needed to characterize this phenomenon. Considering the availability of transgenic mouse models, a standardized protocol for inducing and evaluating LTF in murine models would be extremely advantageous for studying how human genes influence spinally-mediated breathing plasticity.

### Sex effects

Another explanation for the lack of observable differences between genotypes is that they could be masked by sex effects. *APOE* has long been studied in the Alzheimer’s field, where genotype influences are known to be modulated by sex. While expression of the ε4 allele increases the risk of developing AD in both males and females, this risk is greater in females (Duara et al., 1996; Altmann et al., 2014). In rodents, apoE4-related deficits in learning and memory are aggravated in females, indicating that synaptic plasticity in the brain is impaired in a sex-dependent manner (Raber et al., 1998; Kulkarni et al., 2020).The implication for similar trends in spinally-mediated plasticity led to further analysis of our data, in which the influence of genotype was investigated separately in males and females.

Diaphragmatic EMG recordings from females revealed a significant difference between the response of apoE3 and apoE4 animals; 40 min after IH, diaphragmatic activity was significantly depressed in females expressing ε4. Consistent with findings in the brain, this demonstrates that apoE4 females have a limited propensity for plasticity in the spinal cord. This pattern was not reflected in male mice. In contrast with the current body of literature, we show that apoE3 males experience a barrier to synaptic plasticity, as they display the largest decrease in diaphragmatic activity.

To our knowledge, apoE3-associated attenuations of plasticity have never been reported in young adult mice. Since the majority of apoE literature describes its effects on the brain and the periphery, it is possible that our results are because of a unique action of apoE in the spinal cord. The mechanism behind induction of LTF in the bulbospinal breathing circuitry is similar to that of long-term potentiation (LTP) in the hippocampus: both rely on synaptic strengthening brought about by activation of postsynaptic NMDA receptors and signaling through ERK (English and Sweatt, 1997; McGuire et al., 2005; Hoffman et al., 2012). Disparate results from a variety of studies in targeted replacement mice suggest apoE4 can be detrimental or beneficial to LTP depending on brain region, sex, and age (Levi et al., 2003; Kitamura et al., 2004; Trommer et al., 2004; Korwek et al., 2009). Taking this into account, it is less surprising to see that apoE3 also has the potential to augment or impede similar mechanisms of plasticity. This effect may also be dependent on age and region of the CNS.

### The inhibitory PNN and serotonergic presence after C2Hx in targeted replacement mice

Following SCI, there is a dramatic upregulation of inhibitory CSPGs at the site of injury and in denervated targets (Bradbury et al., 2002; Massey et al., 2008; Alilain et al., 2011). Indeed, after dorsal column transections, there is an upregulation of the CSPG-containing PNN around sensory nuclei (Massey et al., 2008) and in previous studies using lateral C2 hemisections, PMNs became further encased by CSPGs and the PNN (Alilain et al., 2011). Despite the abundance of evidence implicating the importance of CSPGs in limiting plasticity, regeneration, and recovery (for review, see Tran et al., 2018); the influence of human genetics (and *APOE* alleles) on PNN structure and neuronal sprouting in the injured spinal cord has never been investigated. However, a study of human brains indicated that apoE4 augments expression of a CSPG known as brevican in the brain of Alzheimer’s patients, which could explain the more extensive staining of PNN observed in E4 mice (Conejero-Goldberg et al., 2015).

Indeed, our findings indicate that apoE4 females exhibit a greater density of the PNN in the ventral horn region containing PMNs after injury. Although PMNS were not discreetly labeled, upregulation of the PNN at the C4 level ipsilateral to injury suggests that deficits in respiratory motor plasticity could be a consequence of the PNN’s numerous influences on CNS function and plasticity. Appearance of the PNNs containing CSPGs during development ends critical periods in which experience-dependent plasticity shapes neural circuitry. Degradation of CSPGs reopens this critical period and restores synaptic plasticity in the adult CNS (Pizzorusso et al., 2002). Following lateral spinal hemisection, increasing densities of CSPG molecules impede calcium diffusion and block action potential conduction in intact axons that are spared by the injury (Hrabetová et al., 2009; Hunanyan et al., 2010). These molecules also create an inhibitory microenvironment that prevents sprouting and regeneration of fibers in the injured spinal cord, including 5-HT fibers that have the potential to enhance functional recovery after experimental SCI (Alilain et al., 2011; Warren et al., 2018; Warren and Alilain, 2019).

Since serotonergic signaling at the level of PMNs is crucial to induction of LTF (Bach and Mitchell, 1996), we quantified 5-HT staining around the putative PMN in spinal cords from E3 and E4 females. Density of 5-HT fibers was higher in E4 females both contralateral and ipsilateral to injury, although this difference did not reach statistical significance on the contralateral side. This indicates that compared with apoE3, apoE4 females may have greater serotonergic innervation of the PMN in the absence of injury. However, additional studies are needed to determine whether females expressing ε4 have greater serotonergic innervation before injury, as well as after C2Hx. Indeed, if this pattern is consistent regardless of injury status, increased 5-HT fiber density could represent a compensatory mechanism that maintains motor neuron excitability in these animals while combatting the loss of synaptic integrity over time that is observed in E4 animals (Klein et al., 2010). The observed attenuation of LTF after injury may therefore be because of apoE4-dependent decreases in 5-HT receptor expression on PMNs, or a result of alterations downstream of 5-HT receptor activation in the signaling pathways that are necessary for the induction of LTF.

Although the higher density of PNN in E4 females is not associated with a decrease in the amount of 5-HT at the level of the PMN after injury, the CSPG-containing PNN could still play a role in abrogating respiratory motor plasticity. Further investigations are needed to determine whether CSPGs block ion flow in spared axons such as the CPP after cervical hemisection similar to the inhibition observed after thoracic injury (Hrabetová et al., 2009; Hunanyan et al., 2010). Previous studies have shown that degradation of CSPGs leads to increased presence of 5-HT around PMNs, which is associated with recovery of breathing function. However, these studies did not address at the effect of CSPG upregulation or degradation on glutamatergic sprouting or regeneration (Alilain et al., 2011; Warren et al., 2018; Warren and Alilain, 2019). Therefore, alterations in glutamatergic innervation of PMNs could also contribute to the enhancement of diaphragmatic function demonstrated in these studies. Although E4 females displayed more 5-HT fibers than E3 females, further examination of glutamatergic axon regeneration and sprouting, as well as how enzymatic degradation of CSPGs alters this innervation, could provide insight into whether PNN upregulation contributes to a lack of respiratory motor plasticity in females expressing ε4.

The primary goal of this study was to investigate the role of genetic variability in determining an individual’s propensity for spinal plasticity and recovery of breathing function after SCI. Preclinical studies typically test therapeutic approaches in a homogenous group of animals, which does not represent the diversity found in the human population. As IH and other therapeutics enter clinical trials, their efficacy for treating a heterogeneous population is an important consideration. Overall, our findings that sex and *APOE* genotype modulate the response to therapeutic IH, along with the current dearth of successful treatment strategies for SCI, emphasizes the importance of advancing personalized medicine to improve outcomes for injured individuals.

## Synthesis

Reviewing Editor: Deanna Smith, University of South Carolina Decisions are customarily a result of the Reviewing Editor and the peer reviewers coming together and discussing their recommendations until a consensus is reached. When revisions are invited, a fact-based synthesis statement explaining their decision and outlining what is needed to prepare a revision will be listed below. The following reviewer(s) agreed to reveal their identity: NONE.

Both reviewers agree this work explores a novel idea and addresses an important question. They also agree that the manuscript is well written, and that most of the experiments are well designed. However, there was some concern about the low number of animals used in some experiments. It was suggested that a power analysis be performed to determine if the number of animals is sufficient to draw conclusions about the level of significance. It was also suggested that data on the impact of injury in wild type control mice and data from uninjured animals should be included for comparison. I have included specific reviewer comments below:

Reviewer 1:

This manuscript presents some very interesting results showing how APOE genotype impacts recovery of diaphragm function after cervical-type spinal cord injury, in particular how sex differentially impacts this recovery in the context of two APOE genotypes using human E3 mice and human E4 mice. This is a highly important and novel area of investigation in the spinal cord injury field. Furthermore, while APOE represents only a single gene of interest, the authors nicely used this as a system to begin to explore the impact of genotype on recovery after spinal cord injury. In addition, studying effects on diaphragm function is highly relevant to spinal cord injury.

While this work is important, exciting and innovative, the authors unfortunately used small animal numbers for the various experimental conditions in several of the studies. This impacts some of the conclusions that can be drawn from these studies. For example, the authors state that the effect of the E3 genotype on the data shown in Figures 2C-D displays a divergence between the two genotypes. I can see this trend; however, they only have an “n” of 6-7 per condition, which - at least in this case - is not sufficient. Why have they not included a greater “n” for each condition? ...especially since they included greater animal numbers for the females in Figure 3 (especially for the E4 genotype). Furthermore, the n’s used for the HVR experiment are very low (i.e. only 2-3 per group). I realize that these HVR results are presented as extended data and are not necessary for their conclusions from the main data, but - like some of the other experiments in this study - the inclusion of a single additional mouse could easily completely change the result. In addition, the WFA data appear to show a strong trend, but again the n’s are low such that the difference is not significant (though it’s p = 0.07). This is not to criticize the importance of this work or to criticize the way these experiments were conducted. Instead, it just seems that the small n’s limit the interpretation of the data and some of the conclusions that can be made.

Another larger comment has to do with the mouse as a model system to study diaphragm functional recovery after cervical hemisection spinal cord injury, especially the effects of IH on LTF. The rat has been the traditional model system for this type of work. This is definitely not to say that the authors shouldn’t use the mouse. In fact, developing the mouse model to explore these types of questions is very important and will provide a powerful experimental tool for the field. However, the authors report quite different effects of IH compared to the rat cervical hemisection paradigm, which should be considered in some of the conclusions. The authors state, “Our results demonstrate that the therapeutic potential of IH is modified by sex and apolipoprotein E genotype.” They should be careful with a conclusion like this, as they don’t observe a therapeutic-type effect of IH in their mouse model. This is not to say that their results aren’t important, as they still observe an interesting impact of the APOE genotype on diaphragm EMGs in response to IH; however, they are not really evaluating the effects of APOE genotype on therapeutic effects of IH. On a more technical/experimental level, is it that they have not determined the appropriate IH paradigm to produce this LTF-like plasticity response in mice (versus rats)? Or is this not even possible in mice? As this mouse cervical injury system has not been used extensively to study diaphragm recovery (and because the authors don’t observe the same LTF response as in rats), it would be helpful to present the EMG data from uninjured mice to compare to the effects of hemisection. Since they don’t observe the expected effects of IH on LTF, it seems like knowing the EMG data in uninjured mice is important. In addition, it would be nice to know if there is any spontaneous recovery (i.e. without IH) over time post-injury after hemisection in this mouse model (and between the two APOE genotypes)? This could involve comparing EMG amplitude in injured mice at different time points post-injury, as well as comparing EMGs in the injured mice at these different time points to uninjured mice. In this study, they conducted the IH and EMG recording at three weeks after injury, and it sounds like the ipsilateral diaphragm was silent (was it completely silent?) in most or all mice. Can they provide the quantification of this EMG, both over time post-injury and compared to uninjured mice.

Reviewer 2:

This manuscript examines an interesting topic “Novel influences of sex and APOE genotype on spinal plasticity and recovery of function after spinal cord injury.” They found that apoE4 in females prevented the augmentation of breathing in response to intermittent hypoxia. As a whole, the paper provides convincing evidence that apoE4 and gender plays a role in respiratory plasticity after SCI. Highlighting the importance of gender and genetics in recovery and therapeutics after SCI. As such, I am pleased to say that I recommend this manuscript to be accepted after addressing the following comments below:

Methods:

Was EMG recorded from both the left and right sides of the diaphragm? Was there a change in diaphragm EMG amplitude in E3 or E4 mice?

Please indicate the timescale of the traces in 2B.

Was the decrease shown in figure 2D significant?

Was the core body temperature measured during the hypoxic ventilatory response?

Line 212: It is stated there was a decrease in the frequency. “As evidenced in these traces, both genotypes exhibited a decrease in frequency over time after IH.” However, the representative traces show a decrease in frequency on APOE4 in figure 2A.

Line 319: “;However, our data demonstrates that mice expressing human apoE isoforms did not differ in their diaphragmatic response to IH, indicating that there may be no effect on LTF when APOE genotype is the sole variable being considered,” Does the presence of the APOE alleles affects the presentation of LTF in the intact state?

Why was three weeks after injury chosen as the time point to examine plasticity after the spinal lesion?

Was the increase in PNN around the phrenic motoneurons? It might be interesting to determine if WFA expression limits the expression of serotonin receptors on the motoneurons?
