# Supplementary figures and images for: Novel Influences of Sex and APOE Genotype on Spinal Plasticity and Recovery of Function after Spinal Cord Injury
Source: eNeuro. 2021 Mar 5;8(2):ENEURO.0464-20.2021. doi: 10.1523/ENEURO.0464-20.2021 (PMC7986541; doi:10.1523/ENEURO.0464-20.2021)

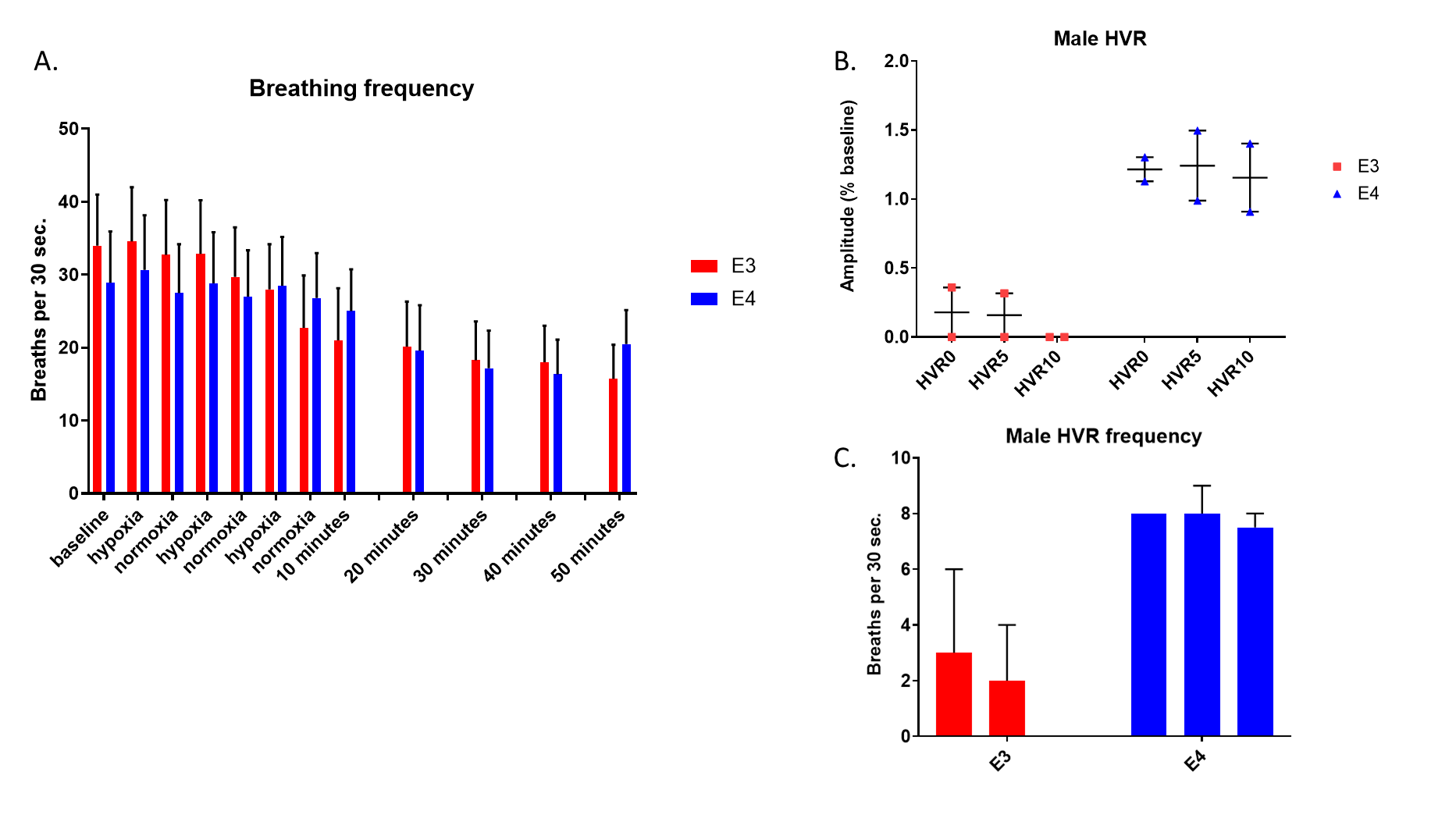

Supplement: Extended Data Figure 2-1 — The respiratory response to hypoxia is determined by APOE genotype in male mice. A, Quantification of diaphragmatic burst frequency in male mice. There is no significant difference between the decreases in apoE3 and apoE4 mice (RMANOVA p = 0.846). B, C, Quantification of diaphragmatic burst amplitude (B) and frequency (C) in response to a 10-min hypoxic exposure. Hypoxia appears to attenuate breathing in apoE3 males. No statistics were performed due to low n; E3 and E4 n = 2. Download Figure 2-1, TIF file. [file enu-eN-NWR-0464-20-s01.tif]

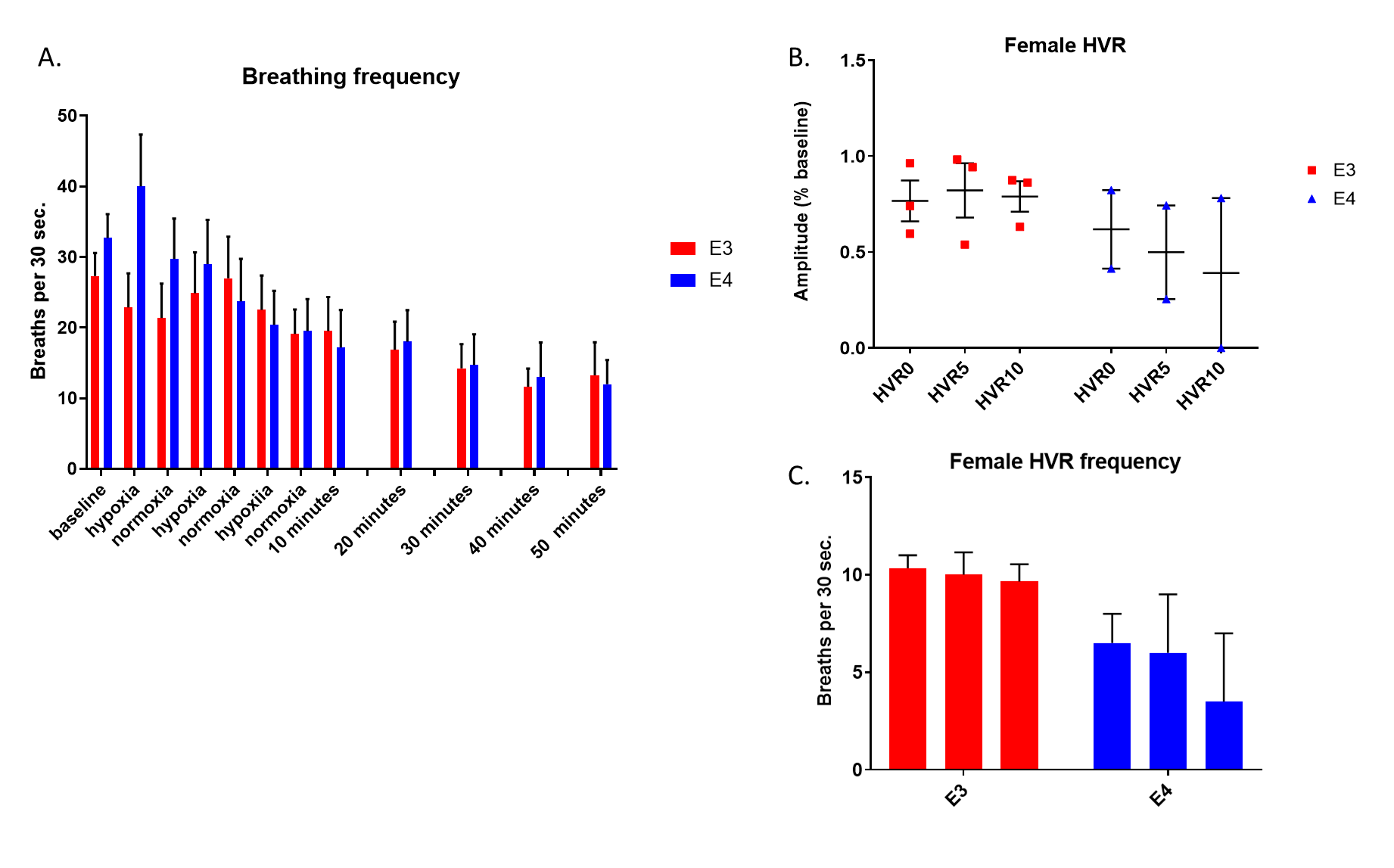

Supplement: Extended Data Figure 3-1 — Hypoxia induces a decline in breathing frequency in female APOE targeted replacement mice. A, Quantification of diaphragmatic burst frequency in female mice. There is no significant difference between the decreases in apoE3 and apoE4 mice (RMANOVA p = 0.673). B, C, Quantification of diaphragmatic burst amplitude (B) and frequency (C) in response to a 10-min hypoxic exposure. Breathing frequency displayed a negative trend in apoE4 females. No statistics were performed due to low n; E3 n = 3, E4 n = 2. Download Figure 3-1, TIF file. [file enu-eN-NWR-0464-20-s02.tif]
